# Supplementary material for: The role of E-cadherin expression in the treatment of western undifferentiated early gastric cancer: Can a biological factor predict lymph node metastasis?
Source: PLoS One. 2020 Apr 29;15(4):e0232429. doi: 10.1371/journal.pone.0232429 (PMC7190119; doi:10.1371/journal.pone.0232429)
Supplement: S1 Fig — (PDF) [file pone.0232429.s001.pdf]

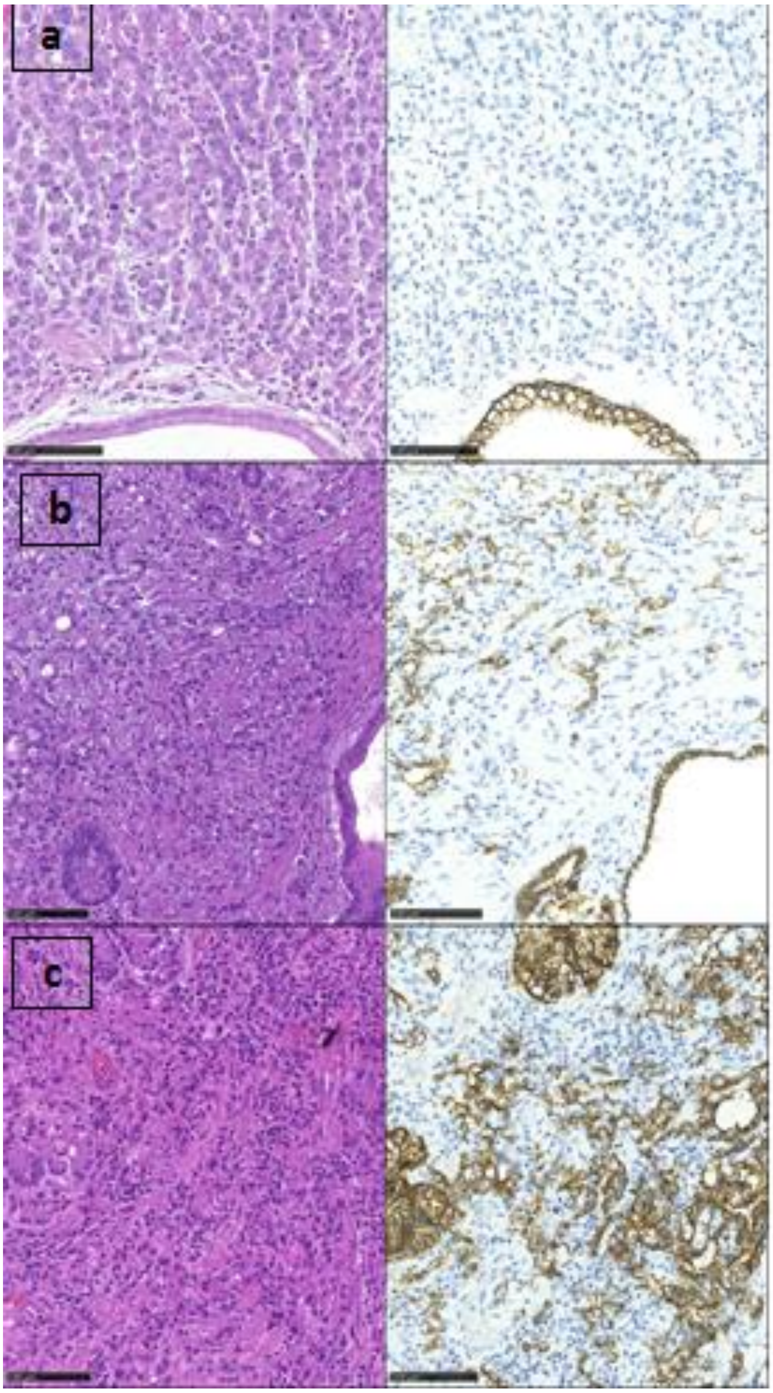

S1 Fig. The E-cadherin (E-cad) expression by immunohistochemical assays (ICH).

a) Absent/low staining, fewer than 10% of tumor cells

b) Moderate staining in only 10-50% of tumor cells

c) Strong staining of more than 75% of tumor cells
