# Supplementary material for: The role of E-cadherin expression in the treatment of western undifferentiated early gastric cancer: Can a biological factor predict lymph node metastasis?
Source: PLoS One. 2020 Apr 29;15(4):e0232429. doi: 10.1371/journal.pone.0232429 (PMC7190119; doi:10.1371/journal.pone.0232429)
Supplement: Attachment — Submitted filename: Response to reviewers.docx [file pone.0232429.s002.docx]

**Response to reviewers**

**Journal Requirements:**

1. *Please ensure that your manuscript meets PLOS ONE's style requirements, including those for file naming.*

We consulted PLOSE ONE’s online submission guidelines and carefully revised the manuscript style to meet the journal requirements.

2. *We suggest you thoroughly copyedit your manuscript for language usage, spelling, and grammar.*

The manuscript was kindly copyedited by natural English speaker: Emanuele Lo Menzo M.D, PhD, FACS, Associate Professor of Surgery, Department of General Surgery, Division of Minimally Invasive and Metabolic Surgery, Cleveland Clinic Florida, Weston, FL, USA.

3. *At this time, we ask that you please provide additional information in your Methods section about the specific methodology used to conduct the immunohistochemical analysis in your study.*

Thank you, we provided additional information about the IHC methodology used for the study and included it in the Methods section of the manuscript.

4. *Please provide an amended Funding Statement that declares *all* the funding or sources of support received during this specific study.*

The authors received no specific funding for this work. We modified our cover letter.

5. *Please include a caption for figure 1.*

Figure 1 along with its caption was submitted as supporting information in the requested style and format.

**Review Comments to the Author**

Reviewer #1: This is an interesting paper on an important topic. How to help to predict the presence of lymph node involvement in early gastric cancer. however authors must refer to prevalence and not to incidence of nodal involvment . Another interesting data is to correlate macroscopic aspect of early gastric cancer and E-cad. Paper must be revisad by native English

Thank you for the suggestion; We recognize our mistake in referring to incidence instead of prevalence and corrected it in the manuscript. The correlation between macroscopic features of EGC and E-Cad expression has been included in the revised manuscript. Paper has been revised by a native English speaker.
